# Two-Rooted Maxillary First Molars with Two Canals: A Case Series

**Published:** 2013-01-20

**Authors:** Sahar Shakouie, Hadi Mokhtari, Negin Ghasemi, Seddigheh Gholizadeh

**Affiliations:** 1 Department of Endodontics, Dental School, Dental and Periodontal Research Center, Tabriz University of Medical Sciences, Tabriz, Iran

**Keywords:** Anatomy, Dental Pulp Cavity, Molar, Tooth Abnormalities, Tooth Root

## Abstract

Thorough understanding of the anatomic and internal morphology of a root canal system is absolutely essential for the success of endodontic treatment. Since permanent maxillary first molars have shown variation in internal anatomy, morphology, this tooth has been reviewed extensively. Presence of two canals in a two-rooted maxillary first molar has rarely been reported in studies describing tooth and root canal anatomies. In this report, three cases are presented, which involve the root canal treatment of maxillary first molars with fusion of the two buccal roots.

## 1. Introduction

Tooth root internal morphology is often complex and greatly influences endodontic treatment. In fact, successful endodontic treatment depends on proper cleaning, shaping, and filling of the root canal system; this implies that inability to detect, debride, and obturate all the existing canals is a major cause of endodontic failure [1, 2, 3].

Maxillary first molars have the most complicated root and canal morphology of the maxillary dentition; therefore, their anatomy has been evaluated extensively in various studies. There is a wide range of variations in the literature with respect to the number of canals in each root and the number of roots. It is now generally accepted that the most common form of maxillary first molar has three roots and four canals [4]; the mesiobuccal root has two root canals due to its wide buccolingual dimension and associated concavities and a single canal for distobuccal and palatal roots [5, 6]. The incidence of two mesiobuccal canals has been reported to range from 18% to 96.1% [7, 8, 9]. Other variations for maxillary first molars include one [9], four [10], and five [11] roots and unusual morphology of root canal systems within individual roots. Cases with five [12] and six [13] root canals or with a C-shaped canal configuration [14] have also been reported earlier.

Martinez-Berna and Ruiz-Badanelli [15] reported three cases in which the maxillary first molars involved six root canals (three in the mesiobuccal, two in the distobuccal and one in the palatal roots). Palatine root canal variations were well established by Stone et al., who reported the endodontic treatment of maxillary molars with two palatal roots [16].

Two-rooted maxillary first molar with two canals has rarely been reported. Such an anatomic variation has been reported in a limited number of studies for second maxillary molar. The present case series reports three maxillary first molars with fusion of the two roots and two canals [17].

## 2. Case Report

Case 1

**Figure 1.**
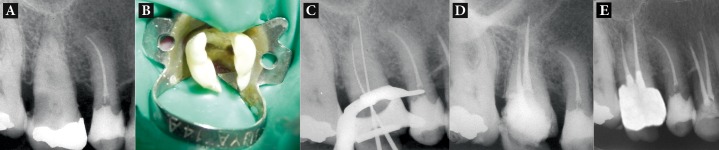
A) Initial radiograph of first maxillary molar with one buccal root; B) Two orifices were detected in the floor of the tooth; C) Working length determination; D) Post-operative radiograph after treatment; E) One-year recall

A 56-year-old female presented to the Department of Endodontics, Tabriz Faculty of Dentistry, with a chief complaint of spontaneous toothache in her maxillary right first molar for the previous two days. The patient’s medical history was unremarkable. The tooth was sensitive to temperature variations and electric pulp test and tender to vertical percussion. The root structure was not clearly demonstrated on radiograph (Figure 1A). The tooth was diagnosed with irreversible pulpitis with apical periodontitis. Local anesthesia was administered with 2% lidocaine and 1:80000 epinephrine (DarouPakhsh, Tehran, Iran) and a rubber dam was placed. After removal of caries the pulp chamber was completely rinsed with normal saline. Exploration of the root canal orifices resulted in finding one buccal and one palatal orifice.

The buccal orifice was relatively large (Figure 1B). No extra orifice was found by further exploration at ×4.5 magnification of prismatic loupes (Zeiss Eyemag Pro S; Carl Zeiss SpA, Arese, Italy) and under dental operating microscope (DOM) (Seiler Revelation, St Louis, MO). The morphology was confirmed by further radiographic examination as initial radiographs were unclear. The root canals were explored with a K-Flexofile ISO20 (Dentsply, Malliefer, Switzerland) and their lengths were determined by a Root-ZX apex locator (Morita, Tokyo, Japan) and confirmed with a periapical radiograph (Figure 1C).

The canals were then further prepared with RaCe rotary files (FKG; Dentaire, La-Chaux-de-Fonds, Switzerland) with 0.04 and 0.06 tapers to 1 mm short of the radiographic apex up to file #35 with 0.06 taper using the crown-down technique. During root canal preparation, irrigation was performed using normal saline, 2.5% sodium hypochlorite solution, and 17% EDTA. The canals were dried with absorbent paper points (Dentsply, Maillefer) and obturated using cold lateral compaction of gutta-percha (Dentsply, Maillefer) and AH26 resin sealer (Maillefer, Dentsply, Konstanz, Germany). Obturation quality was confirmed radiographically (Figure 1D). Access cavity was then sealed with a temporary restorative material. The patient was referred to the Department of Operative Dentistry for restorative treatment.

Case 2

**Figure 2.**
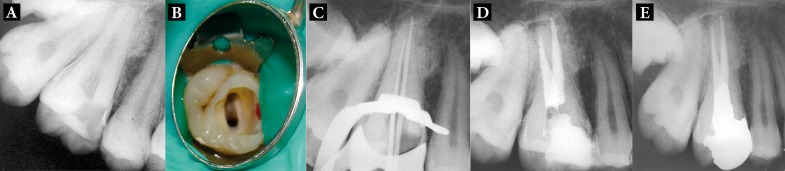
A) Preoperative radiograph of two-canalled first maxillary molar; B) Access cavity reveals one buccal and one palatal canal; C) The master apical cones confirmed the measured lengths; D) Final radiograph; E) 12 month follow-up

A 24-year-old female was referred to the Department of Endodontics, Tabriz Faculty of Dentistry, for endodontic treatment of maxillary right first molar. The pain intensified by thermal stimuli and on mastication. History revealed intermittent pain in the same tooth with hot and cold stimuli for the past two weeks. The patient’s medical history was noncontributory. A clinical examination revealed a carious maxillary right first molar, which was tender to percussion (Figure 2A). Palpation of the buccal and palatal aspects of the tooth did not reveal any tenderness. The tooth was not mobile and periodontal probing around the tooth was within physiological limits. After removal of caries, the roof of the pulp chamber was removed completely and rinsed with normal saline. One orifice was found in the buccal aspect; it had a large diameter compared to typical buccal orifices in maxillary first molars. Then the other orifice was found in the palatal aspect. No other orifice was found even by exploration with a loupe and microscope (Figure 2B). This morphology was confirmed by radiographic examination. The working lengths were determined in the same manner as described for case 1 (Figure 2C); the canals were prepared and obturated as described for case 1. The patient was referred to the Department of Operative Dentistry for restorative treatment (Figure 2D).

Case 3

**Figure 3.**
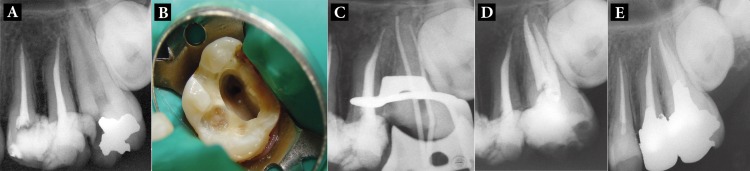
A) Radiographic images of First maxillary molar with two-canal; B) Two orifices were showed in the access cavity image; C) The master cones were inserted at the one buccal and one palatal canal; D) Post-operative radiograph after treatment; E) One-year follow-up

A 32-year-old female was referred to the Department of Endodontics, Tabriz Faculty of Dentistry, for endodontic treatment of her maxillary left first molar. The tooth was sensitive to temperature and electric pulp test but was not tender to percussion. Radiographic examination revealed the presence of a deep amalgam restoration and caries in the mesial aspect (Figure 3A). The root structure was not clearly demonstrated on radiograph (Figure 3A). After removal of the coronal amalgam and caries, access cavity was formed completely and rinsed with normal saline. Compared to typical buccal orifices diameters in maxillary first molars a large buccal orifice was found (Figure 3B). Further exploration was performed using a loupe and microscope, however none were found. The morphology was confirmed by radiographic examination. The working lengths were determined in the same manner as described for case 1 (Figure 3C); the canals were prepared and obturated as described for case 1. The patient was referred to the Department of Operative Dentistry for restorative treatment (Figure 3D). All three cases showed favorable results in one-year follow ups (Figures 1E, 2E, 3E).

## 3. Discussion

The root and root canal morphology of teeth varies greatly according to reported literature [18-28]. Prior knowledge of root and canal anatomy facilitates precise detection of all tooth root canals during endodontic treatment [29]. It has been shown that the total number of canals found and endodontically treated does not correspond to the number of canals actually existing in a tooth. Detection of all the root canals is difficult due to the various factors involved. It is therefore important to understand the variables that affect detection and treatment of root canals. Many studies have evaluated the root and canal morphology of the maxillary first molar because this tooth often presents with complex morphology that often render treatment difficult [4, 29]. Presence of additional root canals has been reported and discussed by several authors, and a variety of study methods, including radiographs, magnification, clinical evaluations, dye injection, tooth sectioning, and scanning electron microscopy have been used for this purpose [29]. Fusion of two buccal roots is one of the most common aberrations of maxillary molars. A total of 0.4% of first maxillary molars and 2.2% of second maxillary molars have been reported to have this variation [30]. This should be considered in endodontic diagnoses and treatments.

Root canal morphology should be examined further during treatment by evaluation of radiographs taken from different horizontal angles. The use of a preoperative radiographs and additional radiographic views with 20-degree mesial or distal angulations are good techniques for the assessment of root canal morphology and anatomy [31, 32].

## 4. Conclusion

Clinicians must have adequate knowledge about root canal morphology and its variations. The location and morphology of root canals should be evaluated by radiography before and during root canal treatment. Careful examination of radiographs and the internal anatomy of teeth are essential for successful treatment.
